# TSS-Seq analysis of low pH-induced gene expression in intercalated cells in the renal collecting duct

**DOI:** 10.1371/journal.pone.0184185

**Published:** 2017-08-31

**Authors:** Yuichiro Izumi, Hideki Inoue, Yushi Nakayama, Koji Eguchi, Yukiko Yasuoka, Naomi Matsuo, Hiroshi Nonoguchi, Yutaka Kakizoe, Takashige Kuwabara, Masashi Mukoyama

**Affiliations:** 1 Department of Nephrology, Kumamoto University Graduate School of Medical Sciences, Kumamoto, Japan; 2 Department of Physiology, Kitasato University School of Medicine, Sagamihara, Japan; 3 Department of Internal Medicine and Education & Research Center, Kitasato University Medical Center, Kitamoto, Japan; Emory University Department of Medicine, UNITED STATES

## Abstract

Metabolic acidosis often results from chronic kidney disease; in turn, metabolic acidosis accelerates the progression of kidney injury. The mechanisms for how acidosis facilitates kidney injury are not fully understood. To investigate whether low pH directly affects the expression of genes controlling local homeostasis in renal tubules, we performed transcription start site sequencing (TSS-Seq) using IN-IC cells, a cell line derived from rat renal collecting duct intercalated cells, with acid loading for 24 h. Peak calling identified 651 up-regulated and 128 down-regulated TSSs at pH 7.0 compared with those at pH 7.4. Among them, 424 and 38 TSSs were ≥ 1.0 and ≤ -1.0 in Log_2_ fold change, which were annotated to 193 up-regulated and 34 down-regulated genes, respectively. We used gene ontology analysis and manual curation to profile the up-regulated genes. The analysis revealed that many up-regulated genes are involved in renal fibrosis, implying potential molecular mechanisms induced by metabolic acidosis. To verify the activity of the ubiquitin-proteasome system (UPS), a candidate pathway activated by acidosis, we examined the expression of proteins from cells treated with a proteasome inhibitor, MG132. The expression of ubiquitinated proteins was greater at pH 7.0 than at pH 7.4, suggesting that low pH activates the UPS. The *in vivo* study demonstrated that acid loading increased the expression of ubiquitin proteins in the collecting duct cells in mouse kidneys. Motif analysis revealed Egr1, the mRNA expression of which was increased at low pH, as a candidate factor that possibly stimulates gene expression in response to low pH. In conclusion, metabolic acidosis can facilitate renal injury and fibrosis during kidney disease by locally activating various pathways in the renal tubules.

## Introduction

Acid-base homeostasis is tightly regulated by interplay between the lungs and kidneys under normal conditions. In the kidney, non-volatile acids taken from food are excreted into urine by collecting duct intercalated cells. As chronic kidney disease (CKD) progresses, metabolic acidosis may occur due to a decrease in acid excretion by the collecting duct. Recent studies have shown that a high protein diet, which is rich in non-volatile acids, is a risk factor for the development and progression of CKD [1–3]. Acid loading accumulates acids and decreases the pH in urine and the kidney interstitium [4, 5]. Metabolic acidosis has been shown to lead to renal hypertrophy and hyperplasia [6]. The administration of sodium bicarbonate attenuates tubular injury [7], thereby delaying the progression of CKD [8]. These findings suggest that a decrease in the pH of urine and the interstitium may affect the function of the renal tubules and possibly cause tubular injury. However, little is known about the molecular mechanisms of acidosis-induced kidney injury. Nowik *et al*. showed that *in vivo* acid loading altered the expression of many genes in the kidney [9]. Cheval *et al*. reported the effect of metabolic acidosis on gene expression in the mouse medullary collecting duct using serial analysis of gene expression (SAGE) [10]. Although these studies suggested that various pathways could be stimulated by metabolic acidosis, the results included both direct and indirect effects of acidosis on heterogeneous cell populations in the kidney. Raj *et al*. performed an *in vitro* microarray analysis and reported that low pH directly stimulated proinflammatory cytokine gene expression in Madin-Darby canine kidney (MDCK) cells [11], a cell line derived from canine distal tubular cells [12]. Because renal tubules are constituted by various differentiated cell types, each type of cell could show a unique response to low pH.

The purpose of the present study was to investigate the direct effects of low pH on the expression of genes that may control local homeostasis in collecting duct intercalated cells. We used transcription start site-sequencing (TSS-Seq) in IN-IC cells, which were derived from the outer medulla of the rat kidney and were characterized as collecting duct intercalated cells [13]. We found many unique genes that were stimulated at low pH. Gene ontology (GO) analysis suggested that the genes could be involved in various mechanisms that promote renal fibrosis. Of note, we found that low pH could locally induce the ubiquitin-proteasome system (UPS), suggesting a pathophysiological role for the UPS in controlling acid-base homeostasis in the kidney.

## Materials and methods

### Cell culture

IN-IC cells that we established in a previous study were used for the present study [13]. Cells were seeded on polystyrene cell culture dishes and fed with DMEM/F12 supplemented with 10% fetal bovine serum (FBS), 10 μg/ml transferrin, 1 μg/ml insulin, 10 ng/ml epidermal growth factor (EGF), 0.5 μg/ml hydrocortisone, 6.5 ng/ml triiodothyronine, and 1% penicillin/streptomycin. All the cells used in the current experiments were at passages 36 to 47. For the TSS-Seq experiments, the cells were preincubated in an experimental solution for 24 h in which the pH was adjusted to 7.4 and were then incubated either at pH 7.4 or at pH 7.0 for an additional 24 h. The experimental solution did not contain FBS or any supplements. The acidity was adjusted by changing the concentration of NaHCO_3_ as previously reported [14]. Two biological replicates were performed. To test the effect of low pH on protein ubiquitination, IN-IC cells were incubated without FBS at pH 7.4 or pH 7.0 for 16 h, and then the cells were treated with 10 μM MG132 (Funakoshi, Tokyo, Japan), a proteasome inhibitor, for either 4 or 8 h. To test the effect of low pH on cell viability, a WST-8 cell proliferation assay was performed following the manufacturer’s protocol (Nacalai Tesque, Kyoto, Japan). After the cells were incubated at pH 7.4 or 7.0 for 24 h, WST-8 was added to the experimental solution. The absorbance was measured at 450 nm after 4 h.

### RNA purification

Total RNA was extracted from the cell lysate using an RNeasy mini kit (Qiagen, Valencia, CA). The RNA quality was assessed with the Agilent RNA6000 Nano Kit (Agilent Technologies, Santa Clara, CA) and standardized at an RNA integrity number (RIN) > 7.0. Quantification via NanoDrop analysis (Thermo Fisher Scientific, Waltham, MA) confirmed that the A260/280 and 260/230 ratios were > 1.7. cDNA libraries for cap analysis gene expression (CAGE) were created [15], and 2 ng of cDNA from each of the libraries was used for sequencing.

### CAGE library preparation and TSS sequencing

CAGE library preparation, sequencing, mapping, gene expression analysis and motif discovery analysis were performed by DNAFORM (Kanagawa, Japan). First strand cDNAs were transcribed to the 5’ end of capped RNAs and were attached to CAGE “bar code” tags. Multiplex deep sequencing of 4 cDNA libraries was performed on an Illumina HiSeq2500 sequencer. The sequenced CAGE tags were mapped to rat reference genomes using the TopHat2 software after discarding the ribosomal and non-A/C/G/T base-containing RNAs [16]. For tag clustering, the CAGE-tag 5’-coordinates were input for RECLU clustering, with a maximum irreproducible discovery rate (IDR) of 0.1 and a minimum tags per million (TPM) value [17], followed by differential expression and motif discovery analyses. The R program edgeR in the RECLU pipeline was used to perform the differential analysis of the genes. GO analysis was performed using the Database for Annotation, Visualization and Integrated Discovery (DAVID) [18]. For motif discovery analysis, clusters with FDR values < 0.05 from the edgeR analysis were used as the foreground sequences, while those with FDR values > 0.4 were used as the background sequences. AMD [19], GLAM2 [20], DREME [21], and Weeder [22] were used to find the *de novo* consensus motifs. The occurrences of motifs were examined by FIMO [23]. The JASPAR database, version 2014 (http://jaspar2014.genereg.net/) was used to search for motif homology.

### Western blotting

Cells were washed twice with phosphate-buffered saline (PBS) and harvested with RIPA lysis buffer. Cell lysates were centrifuged at 16,000 rpm for 10 min, and the supernatants were transferred to new tubes. The protein concentration was measured using BCA protein assay reagents. Proteins were denatured at 95°C for 5 min. Twenty micrograms of total protein were run on a 4–20% polyacrylamide gel (Bio-Rad, Hercules, CA) and transferred to a nitrocellulose membrane (GE Healthcare Life Sciences, Pittsburgh, PA) by electrophoresis. The membrane was incubated in 5% skim milk for 1 h and then incubated with the primary antibody overnight, followed by the secondary antibody for 1 h. The detection of the bands was performed with Enhanced Chemiluminescence Prime (GE Healthcare Life Sciences) using a LAS-4000 mini (Fuji Film, Tokyo, Japan). The ImageJ software was used for the quantitative analysis of protein expression (https://imagej.nih.gov/ij/). The anti-ubiquitin and anti-GAPDH antibodies were purchased from Cell Signaling Technology (Danvers, MA, USA). The anti-H^+^-ATPase B1/2 subunit antibody was purchased from Santa Cruz Biotechnology (Dallas, TX, USA).

### Real-time PCR

mRNA expression was measured by real-time PCR using a TaqMan gene expression assay (Applied Biosystems, Life Technologies, Austin, TX). One hundred nanograms of total RNA were reverse transcribed into cDNA using the Takara PrimeScript RT Master Mix (Takara Bio, Kusatsu, Japan) and amplified using the Eagle Taq Master Mix Kit (Roche Life Science, Indianapolis, IN). The mRNA abundance relative to that at pH 7.4 was calculated as previously published [24].

### Immunohistochemistry

Immunohistochemistry was performed with mouse kidney tissues after acid loading. Either standard rodent chow with 0.4 mol/l HCl added to a ratio of 1 ml/g or water including 0.28 mol/l NH_4_Cl was administered to 10-week-old C57BL/6 mice to induce metabolic acidosis. The kidneys from the control and acid-loaded mice were fixed with 4% paraformaldehyde for 60 min at room temperature or overnight at 4°C. The primary antibodies against H^+^-ATPase, ubiquitin, and aquaporin-2 were used at dilutions of 1:50–500. Anti-aquaporin-2 antibody was purchased from Santa Cruz Biotechnology. Either immunoperoxidase staining or immunofluorescence staining was performed to visualize the antigen-antibody reaction. All animal experiments were evaluated and approved by the Committee for Animal Experimentation at Kumamoto University (A27-144), and the Institutional Animal Care and Use Committee at Kitasato University School of Medicine.

### Statistical analysis

Values are means ± SE. Statistical analysis was performed using analysis of variance (ANOVA) and multiple comparison (Bonferroni or Dunnett’s test) or using the Student’s t-test using GraphPad Prism6 (GraphPad Software Inc, La Jolla, CA, USA). *P* < 0.05 was considered significant.

## Results

### TSS-Seq analysis

**Fig 1:**
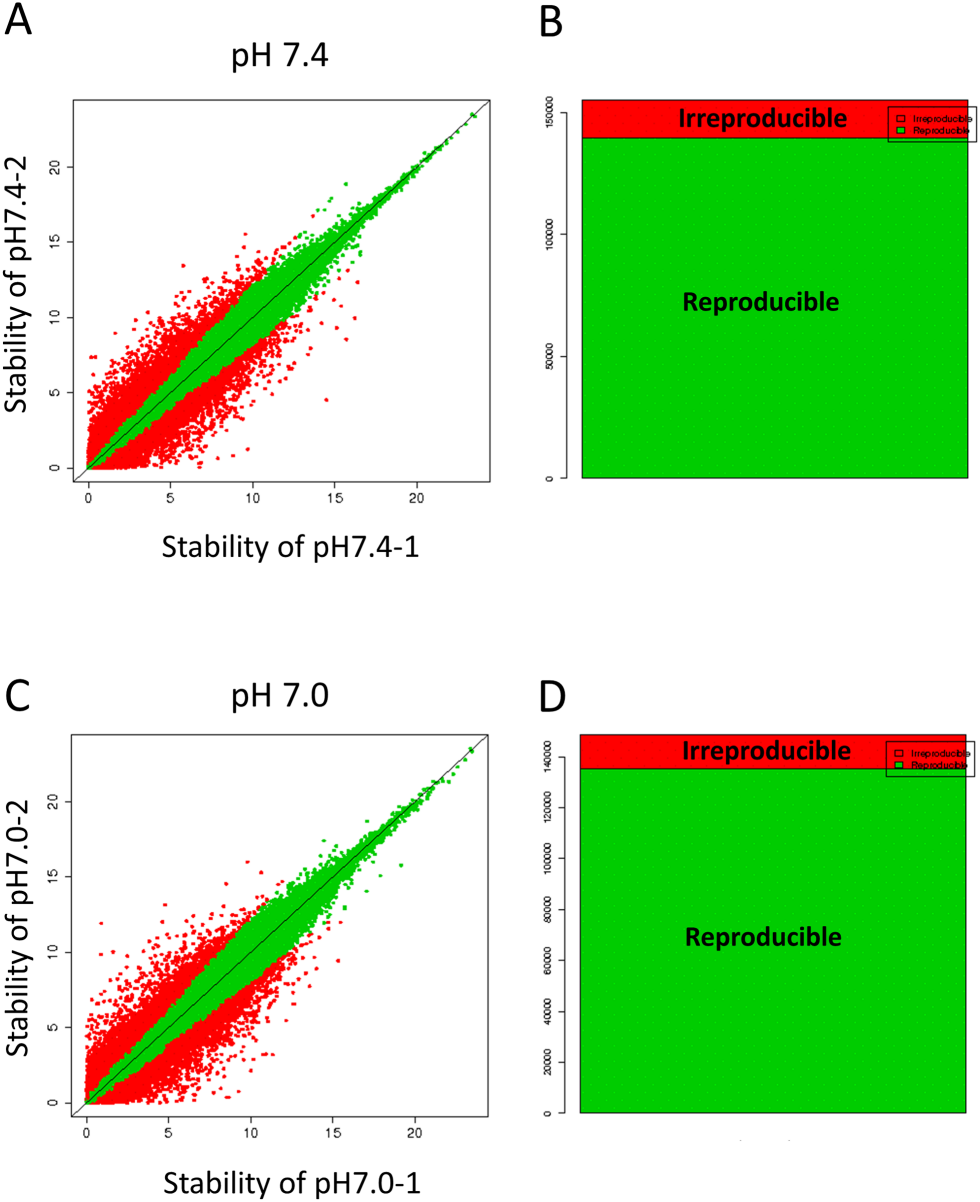
Stability and reproducibility of the biological duplicates. (A and C) The correlations of the expression values between duplicates 1 and 2 at pH 7.4 and pH 7.0, respectively. Green dots indicate high reproducibility and red dots indicate low reproducibility. (B and D) The numbers of reproducible or irreproducible TSS clusters between the duplicates. Green and red bars represent the reproducible and the irreproducible clusters, respectively.

The RIN for each sample is shown in Table 1. More than 17,000,000 reads were obtained from the sequencing of each sample. More than 80% of the reads were mapped to the reference genome (Sequencing/Assembly provider ID: RGSC Rnor_5.0). We confirmed that the expression patterns between the two biological replicates were highly similar (Fig 1). By applying the TSS-Seq analysis, we found 9,963 genes with top peaks and 9,050 genes with bottom peaks. S1 Table includes the list of all the genes with counts per million (CPM) calculated in the peak calling. S2 Table includes the list of genes in which TSSs were significantly up- or down-regulated at low pH. We identified 651 up-regulated and 128 down-regulated TSSs at pH 7.0 compared with those at pH 7.4 (Table 2). Among these TSSs, 424 and 38 TSSs were ≥ 1.0 and ≤ -1.0 in Log_2_ fold change, which were annotated to 193 up-regulated and 34 down-regulated genes, respectively.

**Table 1:** TSS-Seq quality.

Sample name	RIN	Mapped read count	Unmapped read count	rRNA read count	With N read count
pH 7.4–1	8.4	11,281,302	1,509,620	2,092,432	41,589
pH 7.4–2	8.8	11,213,243	1,465,560	1,714,556	40,207
pH 7.0–1	8.5	9,805,544	1,289,155	1,385,315	34,292
pH 7.0–2	8.4	11,675,278	1,635,080	1,949,745	42,756

The quality of the RNA from the samples was evaluated by the RNA integrity number (RIN). Large numbers of reads were mapped to the reference genome.

**Table 2:** Number of up- or down-regulated TSSs and genes by low pH.

Control vs. low pH	Up	Down
Number of significantly changed TSSs (p < 0.05)	651	128
Number of TSSs that showed ≥ 1.0 or ≤ -1.0 in Log_2_ fold	424	38
Number of genes given NCBI Ref Seq IDs among above	193	34

### Low pH-induced gene categories

For the up-regulated genes with expression levels that were increased by ≥ 1.0 in Log_2_ fold change, we used GeneCards (www.genecards.org) to identify the function of the genes (S2 Table). We applied GO analysis to categorize and profile the gene functions (Table 3). Among the genes classified by GO terms that displayed significant enrichment scores, we curated the interesting and relevant genes the roles in the kidney have previously been characterized.

**Table 3:** GO analysis of genes with expression levels that are up-regulated in pH 7.0 (high stringency, enrichment score > 1.5).

Description	Gene ID
***Phosphate metabolic process***	
Protein Tyrosine Phosphatase, Receptor Type, J	Ptprj
Tripartite Motif Containing 28	Trim28
Protein Tyrosine Phosphatase, Receptor Type, A	Ptpra
Cyclin-Dependent Kinase 9	Cdk9
MAP/Microtubule Affinity-Regulating Kinase 3	Mark3
Sperm Associated Antigen 9	Spag9
Polo-Like Kinase 3	Plk3
SH3 Domain Binding Kinase 1	Sbk1
Protein Phosphatase, Mg2+/Mn2+ Dependent, 1J	Ppm1j
Mitogen-Activated Protein Kinase Kinase Kinase 3	Map3k3
Unc-51 Like Autophagy Activating Kinase 1	Ulk1
Glycogen Synthase Kinase 3 Alpha	Gsk3a
Unc-51 Like Autophagy Activating Kinase 2	Ulk2
Mitogen-Activated Protein Kinase Kinase Kinase 1, E3 Ubiquitin Protein Ligase	Map3k1
AP2 Associated Kinase 1	Aak1
Protein Phosphatase 2, Catalytic Subunit, Alpha Isozyme	Ppp2ca
G Protein-Coupled Receptor Kinase 6	Grk6
Protein Kinase, CAMP-Dependent, Catalytic, Alpha	Prkaca
Janus Kinase 2	Jak2
Bromodomain Containing 4	Brd4
Fibroblast Growth Factor 2 (Basic)	Fgf2
***Negative regulation of transcription***	
Nucleus Accumbens Associated 1, BEN And BTB (POZ) Domain Containing	Nacc1
Glutamate-Cysteine Ligase, Catalytic Subunit	Gclc
REST Corepressor 2	Rcor2
YY1 Associated Factor 2	Yaf2
GLIS Family Zinc Finger 2	Glis2
Tripartite Motif Containing 28	Trim28
NGFI-A Binding Protein 1 (EGR1 Binding Protein 1)	Nab1
GATA Zinc Finger Domain Containing 2A	Gatad2a
MDM2 Proto-Oncogene, E3 Ubiquitin Protein Ligase	Mdm2
Nuclear Receptor Corepressor 1	Ncor1
Fibroblast Growth Factor 2 (Basic)	Fgf2
Forkhead Box P4	Foxp4
Akirin 2	Akirin2
***Positive regulation of apoptosis***	
Xeroderma Pigmentosum, Complementation Group A	Xpa
Nucleus Accumbens Associated 1, BEN And BTB (POZ) Domain Containing	Nacc1
CCAAT/Enhancer Binding Protein (C/EBP), Beta	Cebpb
Homeodomain Interacting Protein Kinase 1	Hipk1
Mitogen-Activated Protein Kinase Kinase Kinase 3	Map3k1
Mitochondrial Carrier 1	Mtch1
Transglutaminase 2	Tgm2
Sortilin 1	Sort1
Janus Kinase 2	Jak2
Fas (TNFRSF6) Associated Factor 1	Faf1
***Ribonucleotide binding***	
Septin 3	Sept3
Glutamate-Cysteine Ligase, Catalytic Subunit	Gclc
Ubiquitin-Conjugating Enzyme E2Z	Ube2z
Kinesin Family Member 27	Kif27
Guanine Nucleotide Binding Protein (G Protein), Alpha 11 (Gq Class)	Gna11
ADP-Ribosylation Factor Related Protein 1	Arfrp1
Phosphodiesterase 3B, CGMP-Inhibited	Pde3b
Inositol-Trisphosphate 3-Kinase A	Itpka
ADP-Ribosylation Factor-Like 5A	Arl5a
Mitogen-Activated Protein Kinase Kinase Kinase 3	Map3k3
SH3 Domain Binding Kinase 1	Sbk1
AP2 Associated Kinase 1	Aak1
Mitogen-Activated Protein Kinase Kinase Kinase 1, E3 Ubiquitin Protein Ligase	Map3k1
Transglutaminase 2	Tgm2
Rho-Related BTB Domain Containing 1	Rhobtb1
Protein Kinase, CAMP-Dependent, Catalytic, Alpha	Prkaca
Cyclin-Dependent Kinase 9	Cdk9
MAP/Microtubule Affinity-Regulating Kinase 3	Mark3
Kinesin Family Member 1C	Kif1c
Kinesin Family Member 1B	Kif1b
Polo-Like Kinase 3	Plk3
ATPase, Ca++ Transporting, Cardiac Muscle, Slow Twitch 2	Atp2a2
Glycogen Synthase Kinase 3 Alpha	Gsk3a
Unc-51 Like Autophagy Activating Kinase 2	Ulk2
Phosphatidylinositol 4-Kinase Type 2 Alpha	Pi4k2a
SWI/SNF Related, Matrix Associated, Actin Dependent Regulator Of Chromatin, Subfamily A, Member 5	Smarca5
G Protein-Coupled Receptor Kinase 6	Grk6
Diacylglycerol Kinase, Zeta	Dgkz
Janus Kinase 2	Jak2
ADP-Ribosylation Factor-Like 8A	Arl8a
***Acid-amino acid ligase activity***	
Ubiquitin-Conjugating Enzyme E2E 3	Ube2e3
Glutamate-Cysteine Ligase, Catalytic Subunit	Gclc
Ubiquitin-Conjugating Enzyme E2Z	Ube2z
Mitogen-Activated Protein Kinase Kinase Kinase 1, E3 Ubiquitin Protein Ligase	Map3k1
MDM2 Proto-Oncogene, E3 Ubiquitin Protein Ligase	Mdm2
Ubiquitin-Conjugating Enzyme E2Q Family Member 1	Ube2q1
ubiquitin-conjugating enzyme E2R 2	Ube2r2
***Regulation of protein kinase activity***	
Sperm Associated Antigen 9	Spag9
Mitogen-Activated Protein Kinase Kinase Kinase 1, E3 Ubiquitin Protein Ligase	Map3k1
Diacylglycerol Kinase, Zeta	Dgkz
Protein Kinase, CAMP-Dependent, Catalytic, Alpha	Prkaca
Janus Kinase 2	Jak2
Fibroblast Growth Factor 2 (Basic)	Fgf2
Discs, Large Homolog 1 (Drosophila)	Dlg1
Dishevelled Segment Polarity Protein 1	Dvl1
***Protein catabolic process***	
Xeroderma Pigmentosum, Complementation Group A	Xpa
Ariadne RBR E3 Ubiquitin Protein Ligase 1	Arih1
Ubiquitin-Conjugating Enzyme E2E 3	Ube2e3
Ubiquitin-Conjugating Enzyme E2Z	Ube2z
Microtubule-Associated Protein 1 Light Chain 3 Alpha	Map1lc3a
Ubiquitin Specific Peptidase 9, X-Linked	Usp9x
Mitogen-Activated Protein Kinase Kinase Kinase 1, E3 Ubiquitin Protein Ligase	Map3k1
MDM2 Proto-Oncogene, E3 Ubiquitin Protein Ligase	Mdm2
Ubiquitin Specific Peptidase 25	Ups25
ubiquitin-conjugating enzyme E2R 2	Ube2r2
***Regulation of programmed cell death***	
Xeroderma Pigmentosum, Complementation Group A	Xpa
Nucleus Accumbens Associated 1, BEN And BTB (POZ) Domain Containing	Nacc1
Glutamate-Cysteine Ligase, Catalytic Subunit	Gclc
CCAAT/Enhancer Binding Protein (C/EBP), Beta	Cebpb
Homeodomain Interacting Protein Kinase 1	Hipk1
Mitogen-Activated Protein Kinase Kinase Kinase 1, E3 Ubiquitin Protein Ligase	Map3k1
Mitochondrial Carrier 1	Mtch1
Transglutaminase 2	Tgm2
Sortilin 1	Sort1
Janus Kinase 2	Jak2
Fas (TNFRSF6) Associated Factor 1	Faf1
Fibroblast Growth Factor 2 (Basic)	Fgf2
TNF Receptor-Associated Factor 4	Traf4
Angiopoietin-Like 4	Angptl4
***Positive regulation of cell migration***	
Sperm Associated Antigen 9	Spag9
Insulin Receptor Substrate 2	Irs2
Phospholipase C, Gamma 1	Plcg1
Janus Kinase 2	Jak2
Fibroblast Growth Factor 2 (Basic)	Fgf2
***Regulation of nervous system development***	
Tyrosine 3-Monooxygenase/Tryptophan 5-Monooxygenase Activation Protein, Eta	Ywhah
Serpin Peptidase Inhibitor, Clade F (Alpha-2 Antiplasmin, Pigment Epithelium Derived Factor), Member 1	Serpinef1
Unc-51 Like Autophagy Activating Kinase 1	Ulk1
Unc-51 Like Autophagy Activating Kinase 2	Ulk2
TIMP Metallopeptidase Inhibitor 2	Timp2
Fibroblast Growth Factor 2 (Basic)	Fgf2
Numb Homolog (Drosophila)-Like	Numbl

#### Phosphate metabolic process

**Cdk9** has been reported as a key molecule that promotes renal fibrosis in mice with unilateral ureteral obstructions [25]. **Map3k3** has been shown to activate NFAT5, a transcription factor that induces the expression of osmoprotective genes in response to interstitial hypertonicity [26]. **Ulk1** is involved in autophagy [27]. **Prkaca** has been implied to phosphorylate aquaporin-2 at Ser 256 [28].

#### Negative regulation of transcription

**Gclc** is the catalytic subunit in glutamate-cysteine ligase, which is a ligase involved in the production of the cellular antioxidant glutathione that prevents oxidative stress-induced cell damage [29]. It is transcriptionally up-regulated by Nrf2 and inhibits transforming growth factor-β1 signaling in renal tubular epithelial cells [30]. Loss of **Glis2** induces the growth of cysts caused by nephronophthisis [31].

#### Positive regulation of apoptosis

The expression of **Tgm2** is increased by exposure to advanced glycation end products [32] and is suggested to induce renal fibrosis in the aging kidney [33]. The **Jak2**/Stat signaling cascade is present in tubular epithelial and tubulointerstitial cells and is activated in various renal disease models [34, 35].

#### Ribonucleotide binding

**Gna11** is a calcium-sensing receptor, loss of function mutations of which cause familial hypocalciuric hypercalcemia type 2 [36].

#### Regulation of programmed cell death

**Fgf2** is a key molecule for autophagy that promotes the renal epithelial-mesenchymal transition and interstitial fibrosis [27, 37].

#### Ubiquitin-proteasome System

We identified 18 UPS-related genes that were up-regulated at low pH (Table 4), including 6 ubiquitin protein ligases, 5 ubiquitin conjugating enzymes, 2 deubiquitinating enzymes, 2 ubiquitin-specific peptidases, and three other genes involved in the UPS. Among those genes, 4 ubiquitin conjugating enzymes, 4 ubiquitin protein ligases, and 2 ubiquitin-specific peptidases were classified into GO terms. **Josd1** is a deubiquitinating enzyme that is localized to the plasma membrane [38]. **Mdm2** is a ubiquitin ligase that can interact with 14-3-3γ and degrade UT-A1 [39]. **Siah1** degrades homeo-domain interacting protein kinase 2 (HIPK2), a key molecule in the induction of kidney fibrosis and the epithelial-to-mesenchymal transition in various nephropathies such as human immunodeficiency virus (HIV)-associated kidney disease [40]. **Ube2e3** is a ubiquitin conjugating enzyme that interacts with the E3 ligase Nedd4-2 and regulates ENaC expression in the principal cells of the collecting duct [41].

**Table 4:** UPS-related genes with expression levels that were up-regulated by low pH.

Gene	Description	Function
Angptl4	Angiopoietin-Like 4	Ubiquitin-protein ligase
Ankrd13a	Ankyrin Repeat Domain 13a	Ubiquitin binding protein
Arih1	Ariadne RBR E3 Ubiquitin Protein Ligase 1	E3 ubiquitin-protein ligase
Faf1	Fas(TNFRSF6) Associated Factor 1	Ubiquitin protein ligase binding
Josd1	Josephin Domain Containing 1	Deubiquitinating enzyme
Map3k1	Mitgen-Activating Protein Kinase Kinase Kinase 1	E3 ubiquitin protein ligase
Mdm2	MDM2 Proto-Oncogene	Nuclear-localized E3 ubiquitin ligase
Otud4	OTU Deubiquitinase 4	Deubiquitinating enzyme
Psme4	Proteasome Activator Subunit 4	Associated component of proteasome
Siah1	Siah E3 Ubiquitin Protein Ligase 1	Ubiquitin ligase
Trim28	Tripartite Motif Containing 28	Ubiquitin ligase
Ube2e3	Ubiquitin-conjugating Enzyme E2E3	Ubiquitin conjugating enzyme
Ube2h	Ubiquitin-conjugating Enzyme E2H	Ubiquitin conjugating enzyme
Ube2q1	Ubiquitin-conjugating Enzyme E2E3	Ubiquitin conjugating enzyme
Ube2r2	Ubiquitin-conjugating Enzyme E2R2	Ubiquitin conjugating enzyme
Ube2z	Ubiquitin-conjugating Enzyme E2Z	Ubiquitin conjugating enzyme
Usp25	Ubiquitin Specific Peptidase 25	Ubiquitin specific peptidase
Usp9x	Ubiquitin Specific Peptidase 9, X-Linked	Ubiquitin specific peptidase

#### Effect of Low pH on the ubiquitin-proteasome system

**Fig 2:**
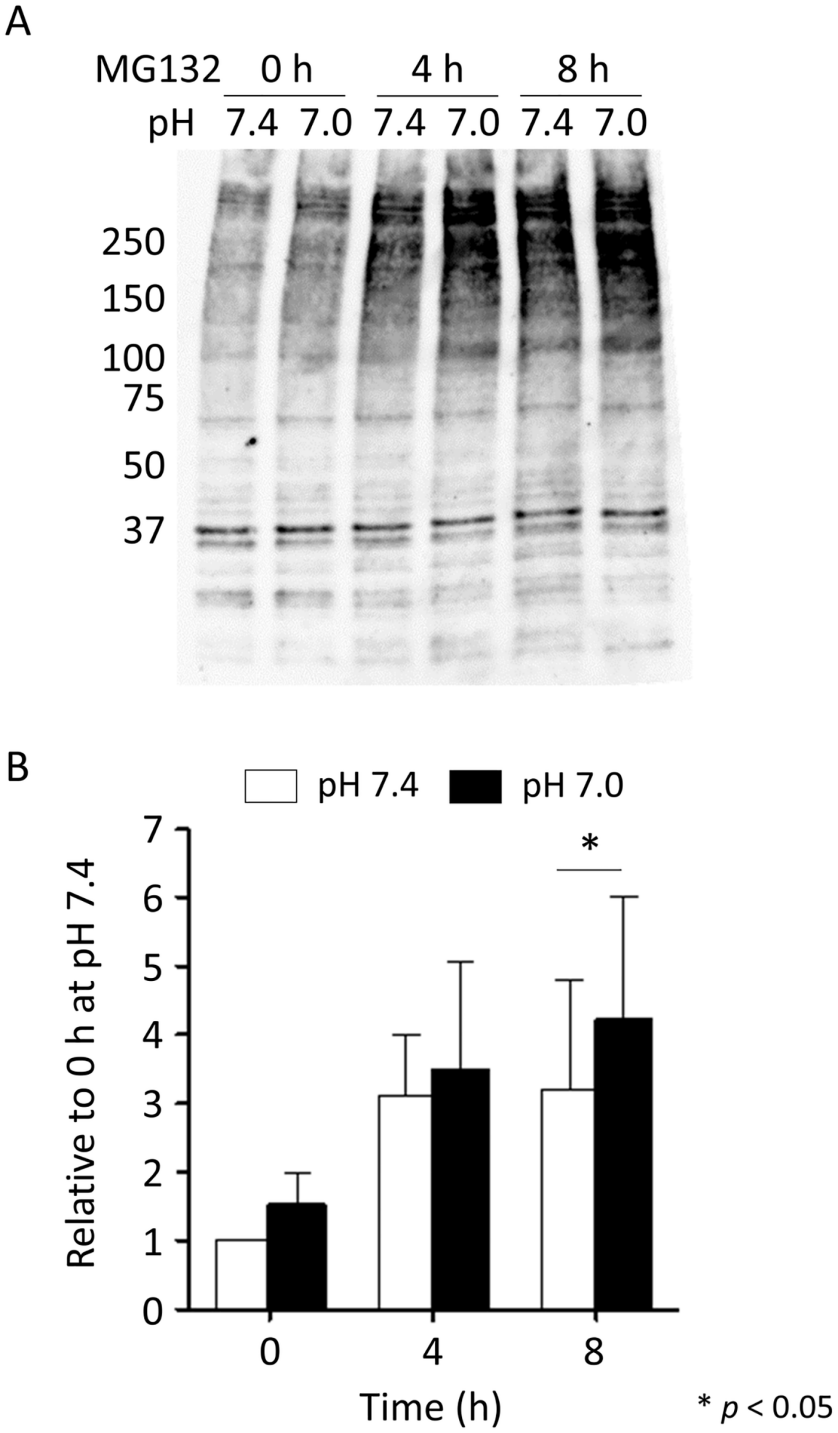
Effect of low pH on the ubiquitination of proteins in IN-IC cells. (A) Representative western blot results for ubiquitinated proteins that were extracted from IN-IC cells incubated at pH 7.4 or at pH 7.0 with MG132. (B) Quantitative analysis of the ubiquitinated proteins by western blot. Band densities higher than 75 kDa were measured. n = 4.

Because the analysis suggested the activation of the UPS, we used western blot analysis to evaluate UPS activity. Western blotting showed an increase in the expression of ubiquitinated proteins in the cells treated with MG132 in a time-dependent manner (Fig 2). The expression of ubiquitinated proteins was greater at pH 7.0 than at pH 7.4, suggesting that low pH activates the UPS.

### Gene expression examined by real-time PCR

**Fig 3:**
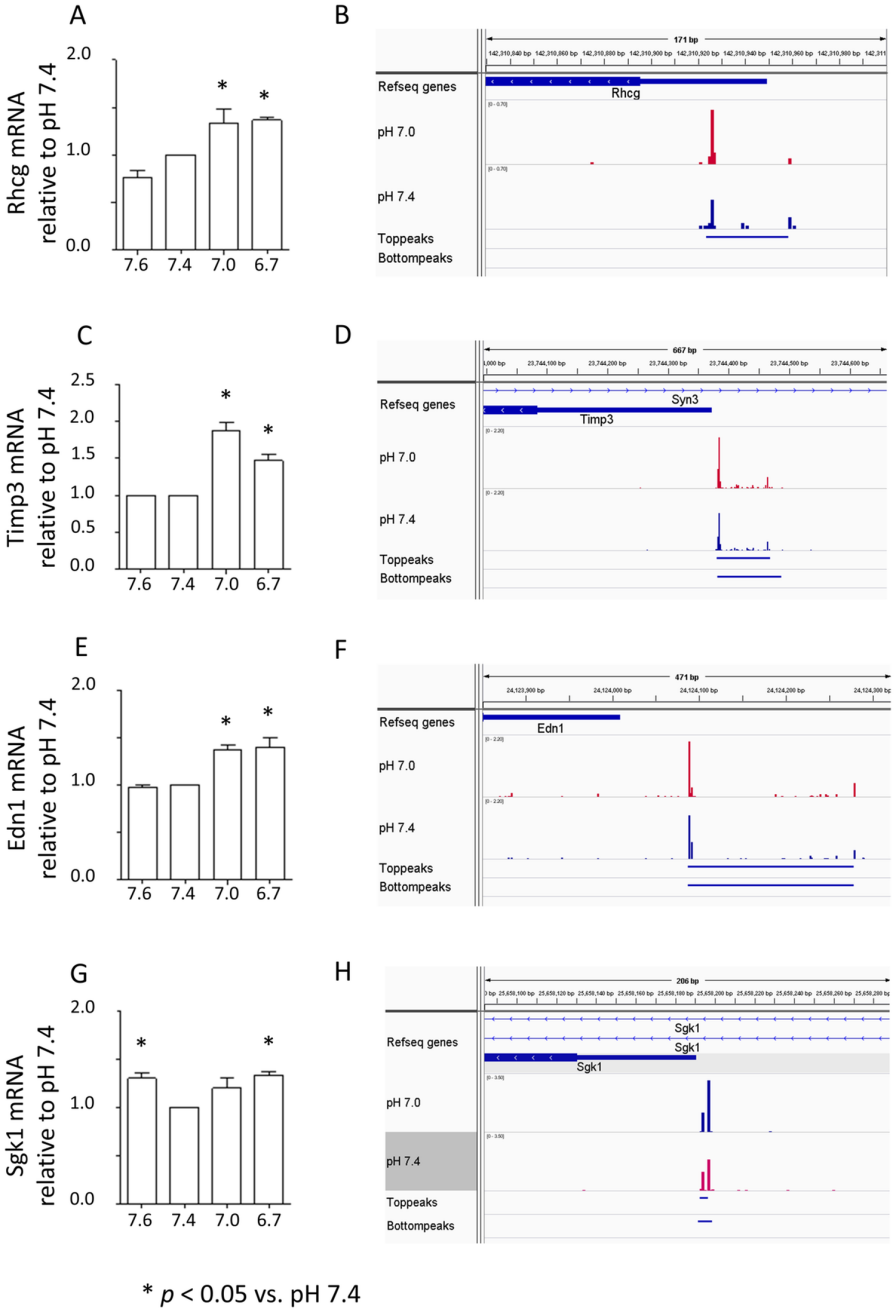
Gene expression levels evaluated by quantitative real-time PCR and visualization of the TSSs of the genes. *p < 0.05 vs. pH 7.4 in 3A, C, E, and G.

Because our TSS-Seq analysis did not show significant changes for some of the genes that were reportedly increased by acid loading *in vivo* in previous studies, we used real-time PCR to analyze the expression of these genes. The expression levels of mRNAs for rhesus blood group-associated C glycoprotein (Rhcg), tissue inhibitor of metallopeptidase 3 (Timp3), endothelin 1 (Edn1), and serum/glucocorticoid regulated kinase 1 (SGK1), the expression levels of which were up-regulated in the outer medulla collecting ducts dissected from mice with NH_4_Cl loading [10], were measured after cells were incubated at pH 7.6, 7.4, 7.0, and 6.7 for 24 h (Fig 3). The expression levels of Rhcg, Timp3 and Edn1 mRNAs were greater at pH 7.0 and 6.7 than at pH 7.4. The expression of Sgk1 mRNA was significantly greater at pH 7.6 and 6.7 than at pH 7.4, although the changes were small. Visualization of the sequencing reads using the Integrative Genomics Viewer [42] showed that transcription levels for these four genes tended to be greater at pH 7.0 than at pH 7.4, consistent with the results obtained by real-time PCR.

### Motif analysis

**Fig 4:**
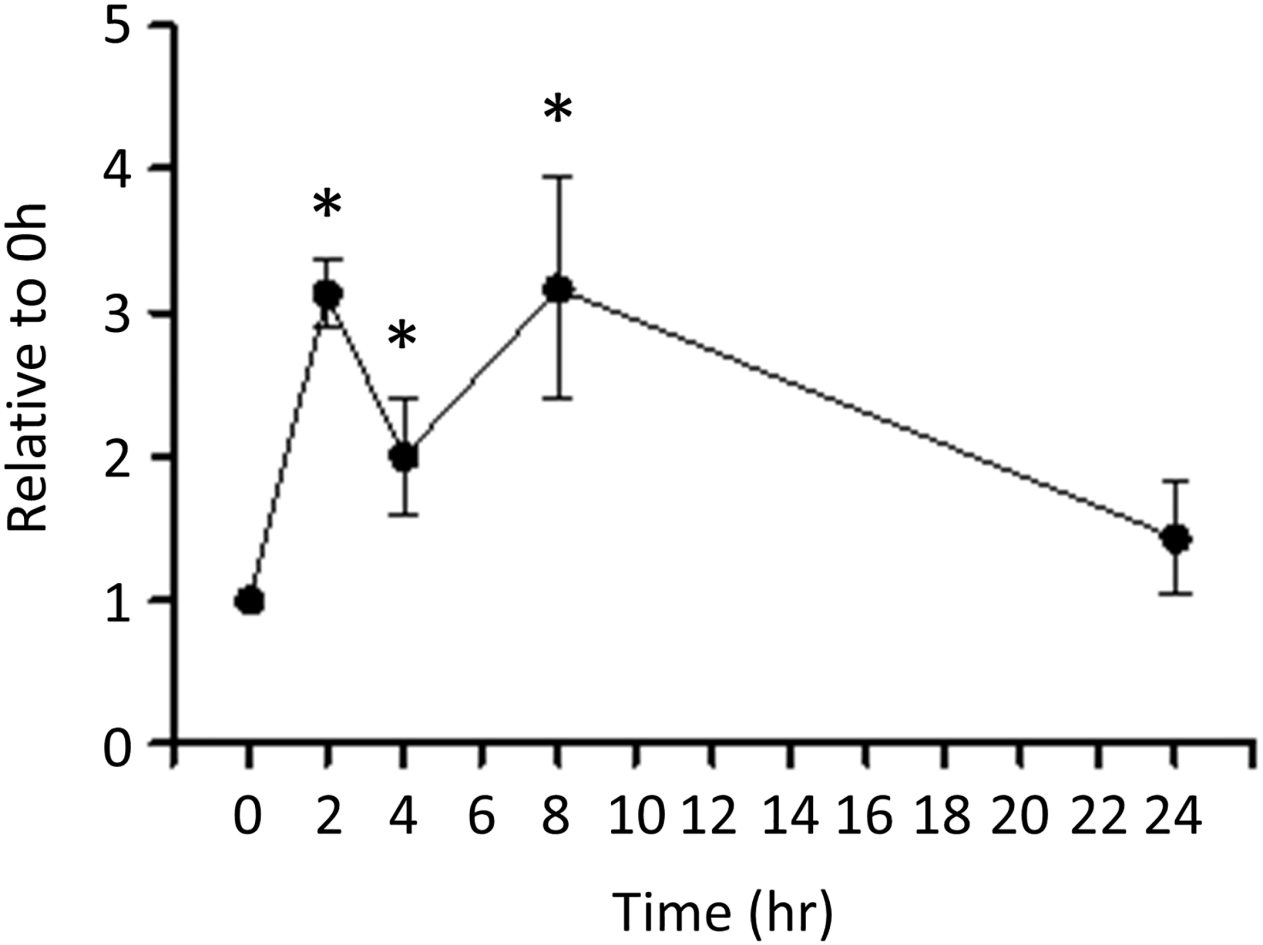
Time-dependent changes in the expression of Egr1 mRNA upon exposure to low pH (pH 7.0) evaluated by real-time PCR. *p < 0.05 vs. 0 h.

Motif analysis was used to search for consensus motifs that could be involved in the regulation of low pH-stimulated genes. S3 Table shows any sequences that possibly coincided with known motifs. A transcription factor is indicated as a target transcription factor (Target ID) when it shows a small p-value and the q-value < 0.05. By merging similar sequences that were possible targets of specific transcription factors, we identified 126 sequences that were significantly enriched in the 5’-flanking regions of genes (S4 Table). Most of the sequences did not correspond to known motifs by searching for homology in the JSPAR database; however, some sequences were similar to Egr1, Egr2, Sp1, and Sp2 motifs (Table 5). We used real-time PCR to confirm that exposure to low pH (pH 7.0) resulted in a rapid increase in Egr1 mRNA expression in IN-IC cells and that its expression remained up-regulated for several hours after stimulation (Fig 4).

**Table 5:** Predicted motif sequences associated with low pH.

Motif no.	Consensus	Foreground	Background	P-value	Known motifs (P-value)
**Up-regulated in top peaks**					
GLAM2_009	GCCBCCGCSCCCGCCCCCGCCCBC	36	2,996	7.33E-03	EGR1 (4.54859e-09) SP1 (5.1807e-06) EGR2 (7.6456e-06) SP2 (9.37891e-06) ZNF263 (2.67598e-05) E2F3 (7.12131e-05)
AMD_009	CMAKSCAGGCCTGARNYY	4	1,299	4.60E-03	Zfx (6.973e-05)
**Up-regulated in bottom peaks**					
GLAM2_009	GVRGSNGSGGCSGGGGGMGGGGGSGGCGBGG	80	3,341	6.59E-05	SP2 (5.97832e-07) SP1 (1.60588e-06) EGR1 (2.09958e-06) ZNF263 (1.87841e-05) E2F3 (2.23805e-05) KLF5 (9.67166e-05)
AMD_001	GGSGGCGGSGGSGGSGGCGGSGGSG	187	2,761	1.48E-31	EGR1 (8.38472e-07) SP2 (6.57943e-05)
AMD_001	GSSGGCGGSSGSGGSGGCGGSGGSG	91	2,805	4.94E-14	EGR1 (1.11672e-06) SP2 (3.44076e-05)
GLAM2_010	GVRGSNGSGGCSGGGGGMGGGGGSGGCGBGG	80	1,137	-1.31E-03	SP2 (5.97832e-07) SP1 (1.60588e-06) EGR1 (2.09958e-06) ZNF263 (1.87841e-05) E2F3 (2.23805e-05) KLF5 (9.67166e-06)
**Down-regulated in top peaks**					
GLAM2_010	SCMCYGCCCCDKSCCCBCYSSGVC	8	2,808	9.99E-03	SP2 (6.00232e-06) EGR1 (1.00592e-05) SP1 (2.43372e-05) KLF5 (3.56756e-05)
AMD_002	ATGNNMTTGNR	14	1,079	5.72E-05	Nr5a2 (3.84899e-05)
**Down-regulated in bottom peaks**					
AMD_006	AAACNKCCNNNNNNCCC	17	1,166	1.26E-06	Pax4 (4.36769e-05)
GLAM2_005	SCCCYCCTSCYCSCYCDCC	28	2,906	5.85E-03	ZNF263 (6.70939e-09) EGR1 (4.27721e-05) SP2 (6.47468e-05)

### Effect of low pH on cell viability

**Fig 5:**
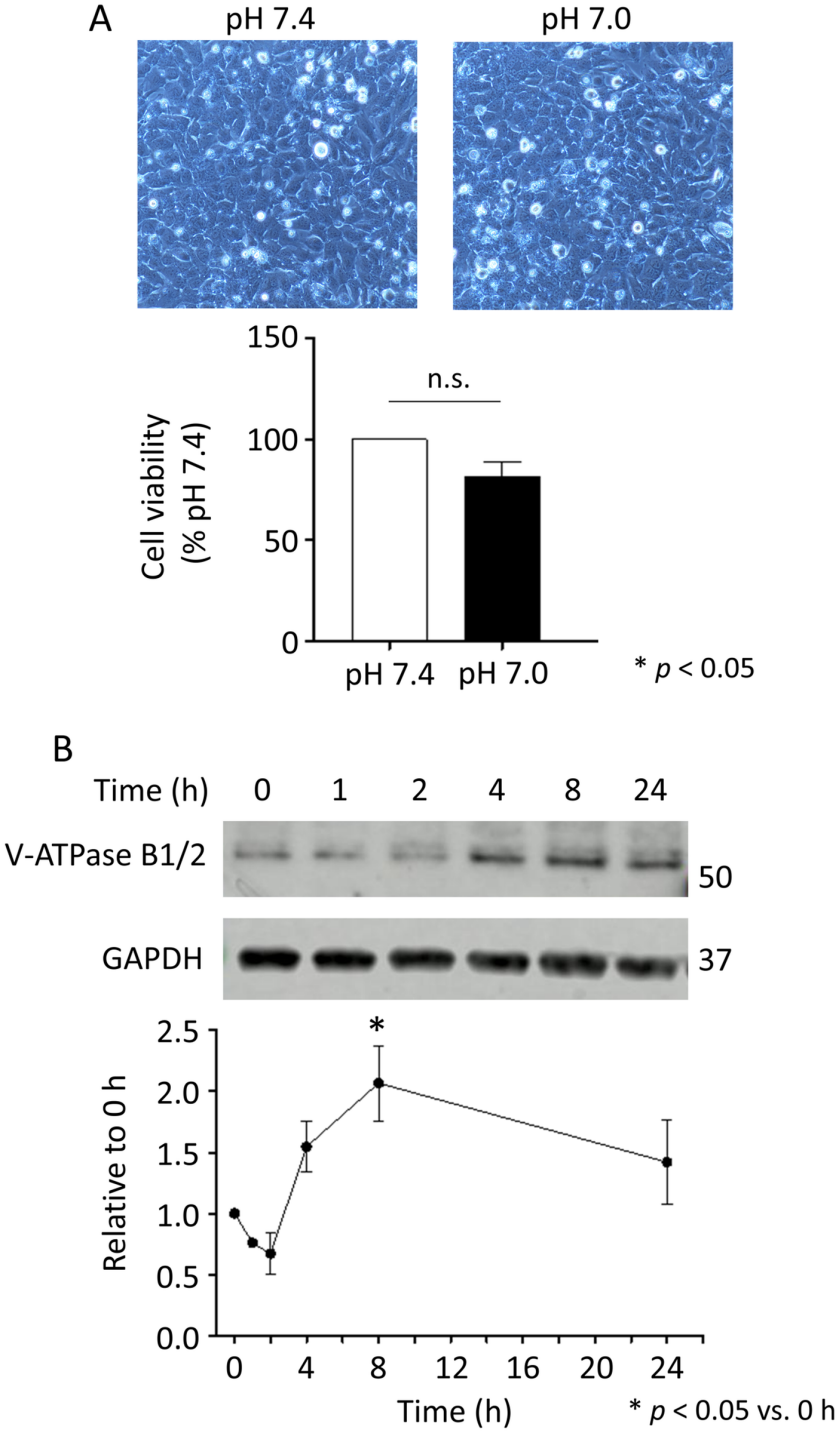
Effect of low pH on cell viability. (A) Cell morphology at pH 7.4 or at pH 7.0 is shown in the upper part. The lower part shows the result of cell viability evaluated by WST-8 assay. (B) Time-dependent change in the expression of H^+^-ATPase B1/2 protein upon exposure to low pH (pH 7.0) evaluated by western blotting.

Because low pH was suspected to cause cell death through the activation of apoptosis and UPS, we examined cell viability after incubation at low pH. Morphological changes were not observed after incubation at pH 7.0 (Fig 5A). The WST-8 cell proliferation assay showed a small decrease in cell viability at low pH, but nothing significant. The expression of H^+^-ATPase B1/2 protein tended to decrease by 2 h at low pH, and then it significantly increased by 8 h. These results suggest that the cells are active and viable at low pH by activating the acid secreting mechanism. pH-induced transcriptional changes could be the precursor of more extensive remodeling.

### Effect of acid loading on UPS in mouse kidneys

**Fig 6:**
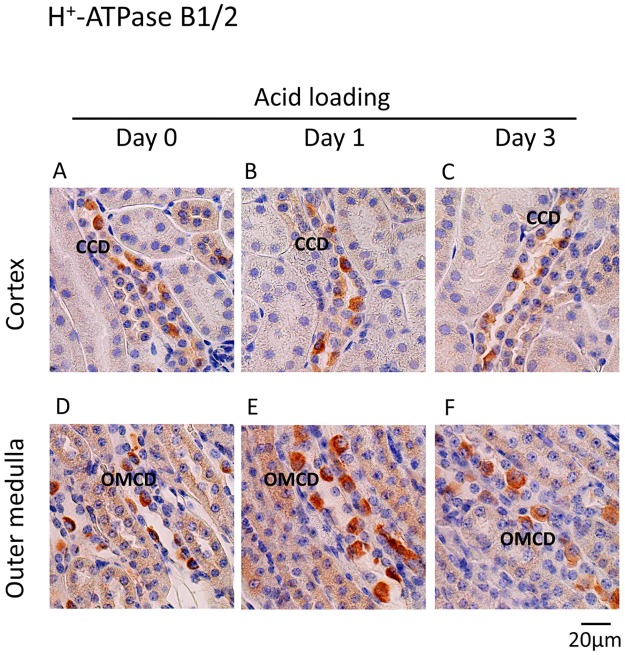
Immunoperoxidase staining of H^+^-ATPase B1/2 in the cortex and outer medulla in HCl-loaded mouse kidneys. Mouse kidneys were extracted (A and D) before and (B and E) one and (D and F) three days after HCl loading. The expression of H^+^-ATPase B1/2 in the cortex and outer medulla was examined. CCD: cortical collecting duct and OMCD: outer medullary collecting duct.

**Fig 7:**
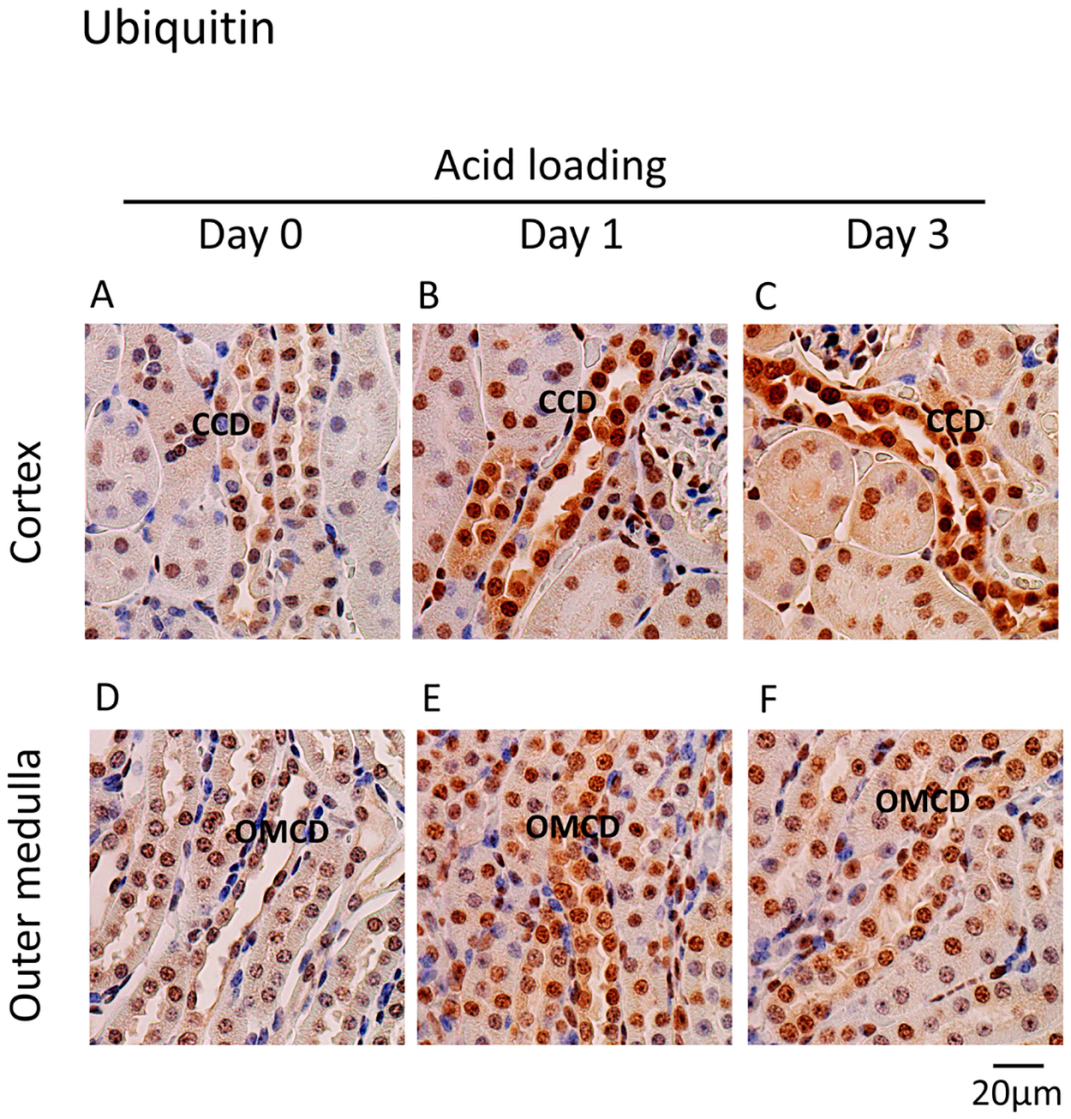
Immunoperoxidase staining of ubiquitin in the cortex and outer medulla in acid-loaded mouse kidneys. Mouse kidneys were extracted (A and D) before and (B and E) one and (D and F) three days after HCl loading. The expression of ubiquitin in the cortex and outer medulla was examined. CCD: cortical collecting duct and OMCD: outer medullary collecting duct.

**Fig 8:**
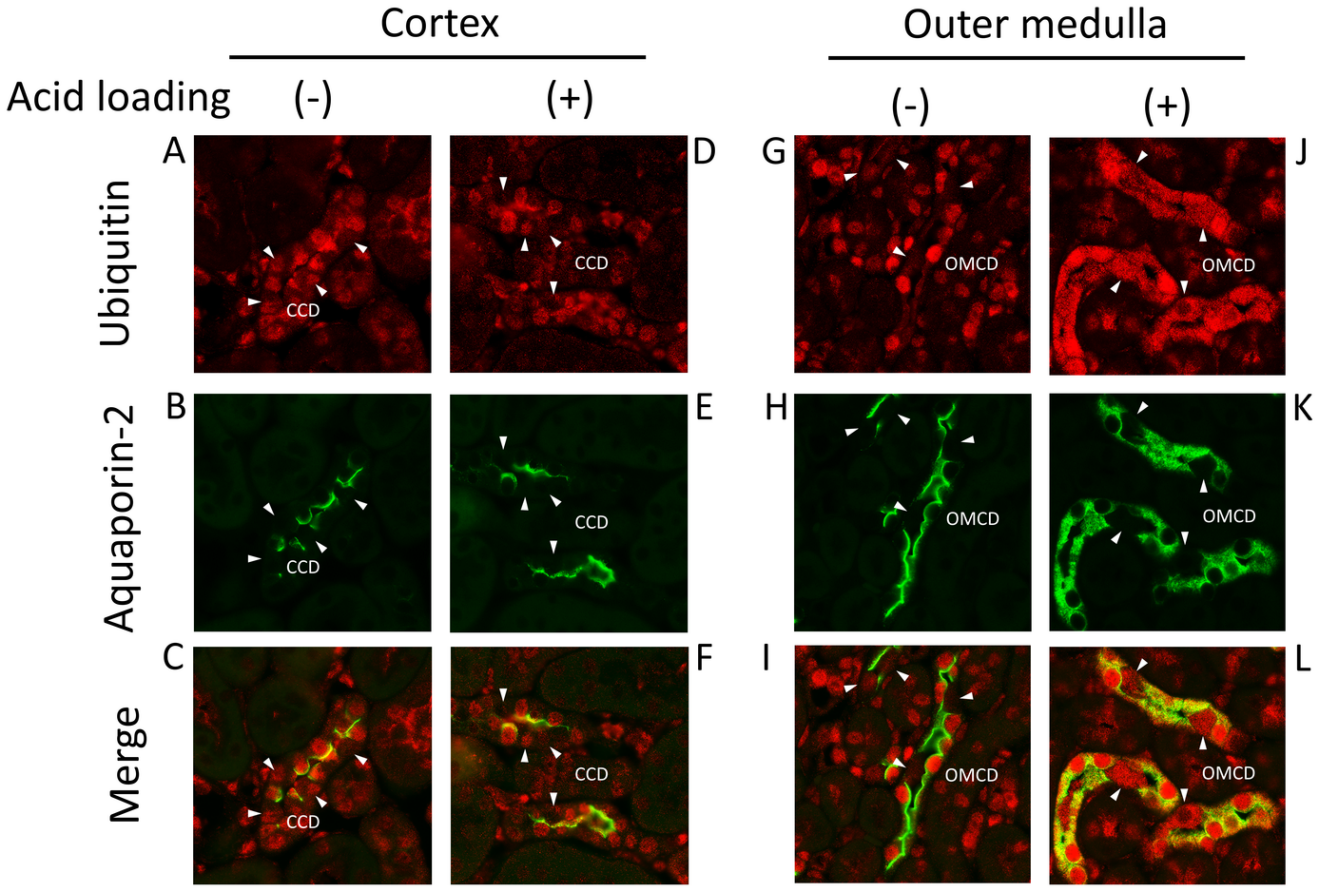
Immunofluorescence staining of ubiquitin and aquaporin-2 in the collecting duct cells in acid-loaded mouse kidneys. Mouse kidneys were extracted (A, B, C, G, H, and I) before and (D, E, F, J, K, and L) three days after NH_4_Cl loading. The expression of ubiquitin and aquaporin-2 in the cortex and outer medulla was examined. White arrow heads indicate aquaporin-2-negative cells, which indicates the intercalated cells. CCD: cortical collecting duct and OMCD: outer medullary collecting duct.

To test the effect of acid loading on UPS *in vivo*, either HCl or NH_4_Cl were loaded into mice and the protein expressions of H^+^-ATPase B1/2 and ubiquitin were examined by immunohistochemistry. H^+^-ATPase B1/2 was mainly expressed in some cells in the cortical collecting ducts (CCDs) and outer medullary collecting ducts (OMCDs) under basal conditions (Fig 6A and 6D). HCl loading increased the expression of H^+^-ATPase B1/2 and caused the hypertrophy of cells expressing H^+^-ATPase B1/2, suggesting that the cells are intercalated cells (Fig 6B, 6C, 6E and 6F) [43]. Ubiquitin was expressed in the nucleus of cells along the nephron, but not in the peritubular cells, under basal conditions (Fig 7A and 7D). HCl loading largely increased expression in the cytoplasm and the nucleus in the CCD and OMCD cells (Fig 7B, 7C, 7E and 7F). Double staining with anti-aquaporin-2 antibody revealed that higher ubiquitin protein expression in the principal cells than in intercalated cells under basal conditions (Fig 8A, 8C, 8G and 8I). NH_4_Cl loading increased ubiquitin expression in both the principal and the intercalated cells (Fig 8D, 8F, 8J and 8L). The localization of aquaporin-2 protein at the apical membrane was inhibited by NH_4_Cl loading, consistent with a previous report (Fig 8B, 8E, 8H and 8K) [44]. These results suggest that the activation of UPS by metabolic acidosis is physiologically relevant.

## Discussion

Metabolic acidosis, a common complication of CKD, has been postulated to exacerbate renal injury. Recently, several clinical studies have suggested that treatment of metabolic acidosis may slow the progression of CKD; some *in vivo* studies have also addressed the pathways for how acidosis may be related to kidney injury [8, 7, 45, 46]. Due to the consumption of acid-enriched diets, renal tubules are exposed to an acidic environment composed of low urinary and interstitial pH [4] [5]. Less is known, however, about the mechanisms of the direct effects of low pH on renal tubular cells. In the present study, we performed TSS-Seq analysis using IN-IC cells to explore the direct and comprehensive effects of low pH on the intercalated cells of the collecting duct. The analysis has shown that many genes are stimulated by exposure to acid. The activation of various biological processes and molecular pathways including the UPS was revealed, implying potential mechanisms relevant to CKD progression caused by metabolic acidosis. Furthermore, the analysis identified a considerable number of genes potentially involved in the process of renal fibrosis, suggesting that acidosis may directly exacerbate kidney injury though the activation of multiple pathways in renal tubular epithelial cells.

We curated the UPS-related genes (Table 4). By searching a database on the RNA-sequencing analysis of microdissected rat kidney tubule segments generated by Knepper’s laboratory [47], we confirmed that 13 of the 17 genes listed in Table 4 are expressed in the cortical collecting duct, where intercalated cells are mainly distributed. In CKD patients, the UPS has been suggested to play a role in muscle wasting and cachexia [46]. It has also been shown that the development of metabolic acidosis in rats with CKD causes a reduction in muscle protein stores due to the stimulation of protein breakdown [48, 49]. Because the major mechanism for the degradation of muscle protein is through the UPS [50], these findings may imply the significance of the UPS in CKD-related complications. Nevertheless, the role of the UPS in the progression of kidney injury remains unclear. Cui *et al*. reported that treatment of OVE26 diabetic mice with MG132 significantly attenuated glomerular and tubular damages by inducing the expression of Nrf2, thereby facilitating downstream antioxidant actions [51]. Immunohistochemical studies with human kidney tissues demonstrated the expression of ubiquitin in Bowman’s capsule parietal cells and tubular epithelial cells [52]. The study also showed that ubiquitin was strongly expressed in tubules under metabolically active conditions such as tubular hypertrophy, while its expression was attenuated in atrophic tubules. In the present study, we demonstrated that ubiquitin protein was largely expressed in the collecting duct cells *in vivo*. Acid loading enhanced this expression. These results suggest that the UPS is pathophysiologically relevant to various conditions in renal tubular epithelial cells.

Regarding the physiological functions of the renal tubule, the UPS may be involved in sodium and water reabsorption in the kidneys. Nedd4-2, an E3 ubiquitin ligase, is known to be essential for regulating epithelial sodium channels (ENaC). Aldosterone inhibits Nedd4-2, resulting in the membrane accumulation of ENaC. In the present study, we did not find an increase in Nedd4-2 transcripts with low pH in IN-IC cells. By contrast, we found an increase in the expression of Ube2e3, a ubiquitin-conjugating enzyme that interacts with Nedd4-2 in principal cells. Additionally, low pH stimulated the expression of Mdm2, an E3 ubiquitin ligase involved in the degradation of urea transporter UT-A1 protein [53]. We have already shown that metabolic acidosis inhibits the accumulation of aquaporin-2 in the apical membrane of the collecting duct [44]. Thus, these findings suggest that acid loading could modulate sodium reabsorption and urine concentration mechanisms through the activation of the UPS in renal tubules.

We also evaluated the effect of acid loading on the expression of some genes the expression levels of which were reported to increase in metabolic acidosis *in vivo*. However, these genes were not significantly changed in our TSS-Seq analysis and showed that there were significant effects of low pH on gene expression levels in IN-IC cells (Fig 3). The results indicate that our analysis extracted genes that have certain responses to low pH. Furthermore, the findings suggest that the expression of the Edn1 and Sgk1 genes, which are known to be up-regulated by aldosterone [54, 55], could be directly stimulated by low pH independently of aldosterone. We also confirmed that treatment with aldosterone increased the expression of Rhcg, Edn1, and Sgk1 mRNAs but not Timp3 mRNA in cells (data not shown). Our study may enable us to dissect pH-dependent and aldosterone-independent mechanisms in the regulation and function of genes expressed in intercalated cells.

Motif analysis revealed Egr1 as a candidate key transcription factor that potentially stimulates the expression of genes in response to low pH (Table 5). We confirmed using real-time PCR that the expression of Egr1 mRNA was increased by acid loading in IN-IC cells (Fig 4). A previous study reported the acid-stimulated expression of Egr1 mRNA in MCT cells, a mouse proximal tubule cell line [56]. Egr1 has been shown to be activated by hypoxia and to induce the epithelial-to-mesenchymal transition in the kidney [57]. A recent report has revealed that Egr1 expression is up-regulated in renal tubular cells in patients with renal failure; in turn, an Egr1 deficiency in mice protects from renal fibrosis induced by an adenine-rich diet [58]. These observations suggest the involvement of Egr1 in acid-stimulated gene regulation in CKD.

In conclusion, we identified a number of low pH-induced genes *in vitro* that are potentially stimulated in the intercalated cells upon metabolic acidosis *in vivo*. The up-regulated genes included a considerable number of genes involved in signaling pathways that promote renal fibrosis. Moreover, we also found that acid loading can activate genes associated with the UPS in renal tubular cells, suggesting a pathophysiological role of the UPS in controlling acid-base homeostasis. Taken together, metabolic acidosis can facilitate renal injury and fibrosis in kidney disease by locally activating various pathways in the renal tubules.

## Supporting information

S1 Table All the genes that were identified by TSS-Seq analysis. All the genes identified in IN-IC cells were listed with counts per million (CPM) calculated in the peak calling.
(XLSX)

S2 Table Differentially expressed genes at low pH. All the genes in which TSSs were significantly up- or down-regulated at low pH were listed.
(XLSX)

S3 Table Sequences that possibly coincided with known motifs. Consensus motifs that could be involved in the regulation of low pH-stimulated genes were searched by the motif-analysis. Any sequences that were possibly coincided with known motifs were listed.
(XLSX)

S4 Table All the sequences that were significantly enriched in the 5’-flanking regions of differential expressed genes. By merging similar sequences listed in S3 Table, we identified 126 sequences that were significantly enriched in the 5’-flanking regions of genes.
(XLSX)
